# The Effect of Anticipated Regret on Flu Vaccination Campaigns

**DOI:** 10.5964/ejop.7749

**Published:** 2023-05-31

**Authors:** Francesco Marcatto, Elisa Detela, Donatella Ferrante

**Affiliations:** 1 Department of Life Sciences, University of Trieste, Trieste, Italy; University of South Wales, Cardiff, United Kingdom

**Keywords:** anticipated regret, flu vaccination, health prevention campaigns, vaccine hesitation

## Abstract

The anticipation of regret is known to be a primary motivator of receiving a vaccination. Aim of this study is to evaluate whether the use of messages that leverage the anticipated emotion of regret can increase the intention to get the flu vaccination. The participants (N = 110) randomly received a leaflet containing a standard prevention message (control condition) or message modified to induce the anticipation of regret over not being vaccinated (experimental condition), along with a questionnaire. The experimental condition’s participants reported significantly higher levels of regret and higher intention to vaccinate than the participants in the control condition. Anticipated regret resulted to be a significant mediator of the intention to get vaccinated. Manipulating the salience of regret appears to be a simple and inexpensive way of effectively promoting preventive behaviour. The implications of this result for reducing COVID-19 vaccine hesitation are discussed.

Research into persuasive health communication repeatedly demonstrates that even small changes in the presentation format of information can have a substantial influence on how people perceive it and, therefore, affect their health-related decisions and behaviours (Waters et al., 2006). For example, people react differently when the consequences of prevention and diagnosis are framed as gains or losses (Gallagher & Updegraff, 2012), when numerical information is presented as natural frequencies or as percentages (Girotto & Gonzalez, 2001), and when the treatment benefits are presented in terms of absolute or relative risk reduction (Marcatto et al., 2013). As a consequence, this knowledge can be greatly useful for designing effective health communication campaigns (Visschers et al., 2009).

The main objective of this study is to evaluate whether health communication messages based on the anticipated emotion of regret can favour the adoption of preventative behaviours, specifically with regards to undergoing influenza vaccination.

Regret is a counterfactual emotion that derives from the comparison between what happened and what could have happened. Since people are regret-averse, this emotion has been found to play an important role in decision-making processes (Marcatto et al., 2015). Indeed, we tend to anticipate how we will feel following a decision and behave in ways that reduce the possibility of experiencing the unpleasant emotion of regret (Mellers et al., 1999). In particular, anticipated emotions such as regret have been found to be immediate precursors of health-related decisions, more so than other relevant factors such as a calculation of the risk probabilities or severity (Chapman & Coups, 2006). For example, in a study on the promotion of safe sex, people who were asked to visualize the negative emotions that they would have felt after engaging in unprotected sex (thus, anticipating regret), tended to subsequently implement greater preventive behaviours than participants in a control group (Richard et al., 1998). Similar effects where preventive behaviours are promoted have been found in different contexts, such as physical activity (Abraham & Sheeran, 2003), the adoption of IT security measures (Wright & Ayton, 2005), and safe driving (Parker et al., 1996).

The anticipation of regret seems to be also a primary motivator of receiving a vaccination (Brewer et al., 2016). In fact, several studies have demonstrated that one of the main factors behind people’s choices on whether or not to have a vaccination is precisely the unpleasant feeling they think they would experience if they did not vaccinate and then were to contract the disease (Chapman & Coups, 2006; Liao et al., 2011; Ziarnowski et al., 2009). For example, in previous research on the factors that predict the outcome of the decision on whether or not to get the flu vaccination, when the researcher interviewed university students before and after having the vaccine, the anticipation of regret was found to be the best predictor among the variables investigated (including concern, the vaccination’s perceived efficacy, gender, the perceived severity of the flu, and the likelihood of getting flu from the vaccine; Weinstein et al., 2007).

However, the studies currently available in the literature have employed correlational or retrospective methodologies alone and, to our knowledge, no intervention studies isolating the effect of changes in anticipated regret on vaccination have yet been published (Brewer et al., 2017).

On this premise, the present randomized study was conducted to investigate the effects of inducing the anticipation of regret on the uptake of the influenza vaccination, not by evaluating its effects retrospectively but by experimentally testing the effects of manipulating the salience of regret on the intention to get the flu shot. Specifically, it was hypothesized that a message based on the anticipation of regret over not being vaccinated is more effective in increasing the intention to vaccinate than a standard message.

## Method

### Procedure

The study was conducted in Autumn 2013, at the beginning of the flu vaccination campaign. Each participant was approached by a research assistant who informed them of the aim of the research, asked if they had already had the flu vaccination, and only in the event of a negative response and consent to participate in the study were they randomized to one of the two experimental conditions (control *vs*. anticipated regret) and did they receive the corresponding booklet. This was handed over with an explicit request to pause and look carefully at the leaflet on the second page before answering the questions.

The study followed the ethical standards laid down in the Declaration of Helsinki and the APA Ethical Guidelines, all measurement instruments were anonymous and participants gave verbal consent to take part in the research. The local public health authority, “Triestina 1”, has given written consent for the use and manipulation of the information material created by them for the purposes of this study.

### Participants

A convenience sample of 110 adults (57 females and 53 males, *M*_age_ = 44.53, *SD* = 14.23) was recruited on public places (public libraries, shopping malls, university campus, etc.).

### Instruments

Participants were presented with one of two versions of a booklet containing a leaflet advertising the flu vaccination along with a questionnaire aimed at evaluating different measures of interest, including the intention to undergo the flu vaccination.

The first page of the booklet presented the study and provided a privacy notice explaining the guaranteed anonymity of the answers provided. The second page contained the leaflet developed by the local public health authority, “Triestina 1”, for the 2013 flu campaign, which depicted a thermometer indicating the temperature of 38°C and a slogan that varied in the two experimental conditions. In the control condition, the slogan was the standard one, “Flu? This year you can avoid it. Get vaccinated!” (“L’influenza? Anche quest’anno puoi evitarla. Vaccinati!”, in the original Italian version). In the regret condition, the slogan read, "Flu? A week off of fever. If only you had been vaccinated..." (“L’influenza? Una settimana di ferie febbre. Se solo ti fossi vaccinato...”, in the original Italian version), with “off” crossed out in red and replaced with “fever”. The leaflets varied only in terms of their message, while the graphics and font used for the slogan were identical.

The third page contained the questionnaire, consisting of six items with answers on a seven-point Likert scale (1 = Not at all, 7 = Very much). The main outcome measures were the intention to vaccinate (operationalized with the item “Are you going to have the flu vaccination?”) and the anticipation of regret (item “If you did not vaccinate and caught the flu, how much would you regret not being vaccinated?”). Further measures collected were the attitude towards vaccines in general (item “How useful do you think vaccines are in general?”), the perception of the effectiveness of the flu vaccine (item “How effective do you think the flu vaccination is in preventing flu?”), the fear of getting sick with the flu (item “How much are you afraid of getting the flu?”), and the perceived likelihood of getting sick (item “How likely do you think you are to get the flu this year?”).

The questionnaire ended with two closed questions in which the participants were asked if, in the previous year, they had fallen ill with the flu, and whether or not they had been vaccinated (Yes/No answers).

### Data Analysis

A power analysis was conducted using G*Power version 3.1.9.7 to determine the minimum sample size required to test the study hypothesis. Results indicated the required sample size to achieve 80% power for detecting a medium effect, at a significance level of .05, was *N* = 102. Thus, the obtained sample size of *N* = 110 is adequate to test the study hypothesis.

A preliminary analysis, using Student’s *t*-test and chi-square test, was conducted to detect significant differences in the demographic composition of the two conditions (control *vs*. anticipated regret). The main analyses of the study consisted in a series of planned Student’s *t*-tests that were run to test the effect of the assigned condition on the main outcome variables. Afterwards, a series of linear regressions were conducted to test whether the anticipation of regret could mediate the effect of the experimental condition on the intention to vaccinate. Bootstrapping procedures with 5,000 samples were used to estimate the indirect effect of the anticipation of regret on the intention to vaccinate and its 95% confidence interval. Finally, a multiple linear regression was conducted with the intent to vaccinate as the dependent variable and demographic variables (age and gender) and all the measures assessed by the questionnaire as possible predictors.

The bilateral level of significance established prior to all the tests was .05. G*Power version 3.1.9.7 was used to run the power analysis, Amos 23 to test the mediation model, and the other statistical analyses were conducted using IBM SPSS Statistics 23.

## Results

The participants of the anticipated regret condition (*N* = 55) did not significantly differ from the participants assigned to the control condition (*N* = 55) in age (*M*_age_ = 43.00 and *SD* = 13.12 in the control condition *vs*. *M*_age_ = 46.05 and *SD* = 15.23 in the anticipated regret condition, *t*(108) = 1.13, *p* = 0.26), gender (28 females in the control condition *vs*. 29 in the anticipated regret condition, χ^2^(1, *N* = 110) = 0.04, *p* = .85), and in the frequencies of having fallen ill with the flu (11 in the control condition *vs*. 18 in the anticipated regret condition, χ^2^(1, *N* = 110) = 2.29, *p* = .13) and having been vaccinated in the previous year (5 in the control condition *vs*. 12 in the anticipated regret condition, χ^2^(1, *N* = 110) = 3.41, *p* = .07).

As shown in Table 1, the participants of the anticipated regret condition reported a significantly greater intention to vaccinate than the participants of the control condition. Similarly, they also reported significantly higher levels of anticipated regret than the participants in the control condition. However, no significant differences emerged in regards to the other measures.

**Table 1:** Comparison of the Main Outcome Variables in the Two Experimental Conditions (t-Test)

	*M* (*SD*)		*t*-test		
Item	Control Condition	Anticipated regret condition	*t* (*df*)	*p*	Cohen’s *d*
Intention to vaccinate	1.96 (1.66)	2.91 (2.46)	-2.37 (108)	.02	0.45
Anticipated regret	2.45 (1.51)	3.22 (2.21)	-2.11 (108)	.04	0.40
Perception of effectiveness	4.22 (1.85)	4.80 (1.77)	-1.68 (108)	.10	0.32
Attitude towards vaccines	4.75 (1.82)	5.31 (1.41)	-1.82 (108)	.07	0.34
Fear of getting sick	2.73 (1.91)	3.07 (2.03)	-0.92 (108)	.36	0.17
Likelihood of getting sick	3.84 (1.95)	3.62 (1.57)	0.65 (108)	.52	0.12

To check whether the effect of the leaflet type on the intention to vaccinate was actually due to a greater salience of the anticipation of regret, a model of mediation of anticipation of regret was tested, between the condition and the intention to vaccinate.

The assigned condition (control condition was coded 0 and anticipated regret condition coded 1) was found to significantly influence both the anticipation of regret (β = 0.20, *p* < .05) and the intention to vaccinate (β = 0.22, *p* < .05). When both the experimental condition and the anticipation of regret were applied as predictors of intent to vaccinate, the latter variable remained significantly associated (β = 0.67, *p* < .001), while the experimental condition did not (β = 0.09, *p* = .21). The indirect effect of the anticipation of regret on the intention to vaccinate was significant (β = 0.13, *p* < .05) and the 95% confidence interval ranged from 0.02 to 0.26, thus suggesting a complete mediation.

Finally, data from the two conditions were aggregated and a multiple linear regression was conducted to assess whether and to what extent it is possible to predict the intent to vaccinate using the following predictors: age, gender (*M* = 0, *F* =1), whether in the previous year the participant had fallen ill with the flu (No = 0, Yes =1), and whether in the previous year they had been vaccinated (No = 0, Yes =1).

The overall regression model explained 64% of the variance in intent to vaccinate and, as reported in Table 2, the only significant predictors were the anticipation of regret, having been vaccinated in the previous year, and the perceived efficacy of the influenza vaccine (*F*(9,109) = 19.77, *p* < .001).

**Table 2:** Multiple Linear Regression (Dependent Variable = Intention to Vaccinate)

Predictors	β	*t*	*p*
Age	-0.03	-0.54	0.59
Gender	0.01	-0.01	0.99
Anticipated regret	0.46	5.38	< .001
Attitude towards vaccines	0.02	0.27	0.79
Perception of effectiveness	0.19	2.06	0.04
Fear of getting sick	-0.03	-0.29	0.78
Likelihood of getting sick	-0.11	-1.43	0.16
Flu in the previous year	0.11	1.59	0.12
Vaccinated in the previous year	0.38	5.53	< .001

## Discussion

The field of health promotion is constantly struggling with new challenges, from the increase of lifestyle-related diseases to ‘vaccine hesitation’, the reluctance of people to receive safe and recommended available vaccines, including COVID-19 vaccines (Machingaidze & Wiysonge, 2021). Communication is at the forefront of the achievement of health promotion objectives, and campaigns represent a primary strategy for public health interventions, acting as a vehicle for health care, public health, and the way society views health. To maximize their potential, campaigns should be developed according to the principles of effective campaign design. Reviews, however, pointed out that most health communication campaigns fail to adhere to these principles (Noar, 2012).

In the present study, we demonstrated that a messages that leverages the anticipated emotion of regret can favour the adoption of a preventive behaviour, such as getting the flu vaccination. Specifically, a leaflet designed to prompt participants to anticipate regret, by making them think at the negative consequences of not getting the flu shot, increased their intention to get vaccinated by about 50% (from 1.96 to 2.91) compared to a leaflet containing a traditional message. Moreover, a mediation analysis showed that this effect is completely mediated by anticipated regret. It was already known that, retrospectively, anticipated regret can explain past decisions, but we showed that it could also be used proactively for designing an effective health communication campaign.

A limitation of this study is that it involved a sample of convenience that was not representative of the older adult population, who are the traditional target of campaigns for the promotion of the flu vaccination. Furthermore, the data was collected before the COVID-19 pandemic so it is possible that people's attitudes towards and interest in the flu vaccination, along with their prevention behaviours, in general, have changed in the meantime. Nevertheless, these limits do not affect the main contribution of this experimental study, namely, that the manipulation of the salience of regret seems to be an effective way to increase the impact of communication in the context of preventative behaviour. Health prevention campaigns could, therefore, adopt this simple and inexpensive mechanism to nudge people towards choices that would allow them to improve their quality of life in terms of their health, including promoting the COVID-19 vaccination. Indeed, although COVID-19 and the seasonal flu differ in many ways, recent research proposed that simple, low cost nudges for boosting the flu vaccination could work for moving the needle on vaccination against COVID-19 too (Milkman et al., 2021). Further studies are, in any case, necessary to evaluate the effectiveness of this approach for preventive behaviours other than those relating to the flu vaccination and to verify the actual implementation of the intentions of the participants.
